# Supplementary material for: Fragment-Based Ligand-Protein Contact Statistics: Application to Docking Simulations
Source: Int J Mol Sci. 2019 May 21;20(10):2499. doi: 10.3390/ijms20102499 (PMC6567162; doi:10.3390/ijms20102499)
Supplement: Supplementary file 1 [file ijms-20-02499-s001.pdf]

Supplementary Materials

Fragment-based ligand-protein contact statistics: application to docking simulations

Gabriele Macari [1], Daniele Toti[1], Carlo Del Moro[1] and Fabio Polticelli[1,2]

[1] Department of Sciences, Roma Tre University, 00146 Rome, Italy
[2] National Institute of Nuclear Physics, Roma Tre University, 00146 Rome, Italy

Screening power

Table S1. Detailed summary of the results obtained in the Screening Power test using different datasets and scoring schemes

	top 1%	top 5%	top 10%
**CS_5003**	1.58	1.32	1.16
**CS_5003avg**	1.41	1.28	1.38
**CS_5005**	1.58	1.44	1.25
**CS_5005avg**	1.92	1.15	1.58
**CS_5007**	0.57	1.03	1.12
**CS_5007avg**	1.92	1.36	1.43
**CS_4003**	1.15	1.32	1.17
**CS_4003avg**	1.92	1.42	1.58
**CS_4005**	1.58	1.55	1.25
**CS_4005avg**	1.41	1.38	1.32
**CS_4007**	0.57	1.15	1.12
**CS_4007avg**	1.92	1.22	1.38
**CS_50avg**	4.61	2.98	2.24
**CS_40avg**	2.56	1.9	1.33

ΔRMSD comparisons

Table S2. Summary of the RMSD comparison. ADV row indicates the number of cases in which the RMSD of the top-ranking pose predicted by AutoDockVina (with respect to the co-crystallized ligand pose) is lower than the RMSD of the CS top ranking pose. For the CS row is the opposite. For Equal the poses predicted by the two approaches have the same RMSD.

	CS_4003	CS_4005	CS_4007	CS_5003	CS_5005	CS_5007
ADV	44	42	46	45	45	45
Equal	19	18	18	19	18	18
CS	22	27	23	22	24	24
